# MRI Evaluation of Axonal Remodeling After Combination Treatment With Xiaoshuan Enteric-Coated Capsule and Enriched Environment in Rats After Ischemic Stroke

**DOI:** 10.3389/fphys.2019.01528

**Published:** 2019-12-19

**Authors:** Man-Zhong Li, Yu Zhan, Le Yang, Xue-Feng Feng, Hai-Yan Zou, Jian-Feng Lei, Ting Zhao, Lei Wang, Hui Zhao

**Affiliations:** ^1^School of Traditional Chinese Medicine, Capital Medical University, Beijing, China; ^2^Beijing Key Lab of TCM Collateral Disease Theory Research, Beijing, China; ^3^Medical Imaging Laboratory of Core Facility Center, Capital Medical University, Beijing, China

**Keywords:** ischemic stroke, *Xiaoshuan* enteric-coated capsule, enriched environment, magnetic resonance imaging, axonal remodeling

## Abstract

*Xiaoshuan* enteric-coated capsule (XSEC) is a compound Chinese medicine widely used for the treatment of ischemic stroke. Enriched environment (EE) is a rehabilitative intervention designed to facilitate physical, cognitive, and social activity after brain injury. This study aimed to assess whether the XSEC and EE combination could provide synergistic efficacy in axonal remodeling compared to that with a single treatment after ischemic stroke using magnetic resonance imaging (MRI) followed by histological analysis. Rats were subjected to permanent middle cerebral artery occlusion and treated with XSEC and EE alone or in combination for 30 days. T2-weighted imaging and diffusion tensor imaging (DTI) were performed to examine the infarct volume and axonal remodeling, respectively. The co-localization of Ki67 with NG2 or CNPase was examined by immunofluorescence staining to assess oligodendrogenesis. The expressions of growth associated protein-43 (GAP-43) and growth inhibitors NogoA/Nogo receptor (NgR)/RhoA/Rho-associated kinase2 (ROCK2) were measured using western blot and qRT-PCR. The Morris water maze (MWM) was performed to evaluate the cognitive function. MRI and histological measurements indicated XSEC and EE individually benefited axonal reorganization after stroke. Notably, XSEC + EE decreased infarct volume compared with XSEC or EE monotherapy and increased ipsilateral residual volume compared with vehicle group. DTI showed XSEC + EE robustly increased fractional anisotropy while decreased axial diffusivity and radial diffusivity in the injured cortex, striatum, and external capsule. Meanwhile, diffusion tensor tractography revealed XSEC + EE elevated fiber density in the cortex and external capsule and increased fiber length in the striatum and external capsule compared with the monotherapies. These MRI measurements, confirmed by histology, showed that XSEC + EE promoted axonal restoration. Additionally, XSEC + EE amplified oligodendrogenesis, decreased the expressions of NogoA/NgR/RhoA/ROCK2, and increased the expression of GAP-43 in the peri-infarct tissues. In parallel to these findings, rats treated with XSEC + EE exhibited higher cognitive recovery than those treated with XSEC or EE monotherapy, as evidenced by MWM test. Taken together, our data implicated that XSEC + EE exerted synergistic effects on alleviating atrophy and encouraging axonal reorganization partially by promoting oligodendrogenesis and overcoming intrinsic growth-inhibitory signaling, thereby facilitating higher cognitive recovery.

## Introduction

Ischemic stroke is one of the most common causes of morbidity and mortality worldwide (Chen et al., 2014). Although stroke mortality has been declining with effective thrombolysis, a large proportion of stroke patients exhibit long-term disability (Zhang and Chopp, 2009). Ischemic stroke induces neuronal loss and elicits profound white matter injury, as characterized by demyelination and axonal injury, which is critical for poor neurological outcomes (Wang et al., 2016; Etherton et al., 2019). Thus, it comes as no surprise that many therapeutic approaches focusing on neuroprotection in rodent models of cerebral ischemia have failed in large clinical trials (Gladstone et al., 2002). Therefore, additional attention should be paid to protect the white matter and boost axonal remodeling that may provide long-term neurological benefits after an ischemic stroke.

*Xiaoshuan* enteric-coated capsule (XSEC) is a Chinese herb compound preparation derived from *Buyang Huanwu* Decoction (BYHWD), a classic traditional Chinese medicinal formula for the treatment of stroke in China for centuries (Hao et al., 2012). BYHWD has shown a convincing effect on neuroprotection and neuroregeneration in stroke patients and experimental stroke animal models (Hao et al., 2012; Zhao et al., 2012; Wei et al., 2013). In particular, it has been reported that BYHWD enhances axonal remodeling and functional recovery after spinal cord injury in rats (Chen et al., 2008) and facilitates axonal regeneration of injured peripheral nerves (Chang et al., 2016; Kim and Namgung, 2018), implicating that BYHWD has a growth-promoting activity on injured axons. However, the unstable quality and lack of uniform standards of BYHWD limits its clinical use. XSEC is a novel preparation of BYHWD approved by the China Food and Drug Administration for treating stroke-induced disabilities (drug permit document: Z20000025). Our previous study demonstrated that XSEC promotes neurovascular remodeling and improves neurological function after ischemic stroke in animal models (Zhang et al., 2016). However, the effects of XSEC on axonal remodeling after stroke have not been investigated.

In recent years, enriched environment (EE) has attracted a great deal of attention in stroke rehabilitation (Janssen et al., 2014; Livingston-Thomas et al., 2016). EE is an intervention designed to facilitate physical, cognitive, and social activity by the provision of equipment and organization in the environment. In particular, many studies have shown that EE promotes the expression of trophic factors and certain transmitters; improves synaptic and axonal plasticity accompanied with reorganization of neuronal networks in the remaining brain after brain injury; and leads to learning, memory, and sensorimotor recovery, either alone or in conjunction with other therapies (Zhang X. et al., 2017; Zhang et al., 2018). Given that EE as a rehabilitative intervention has the potential to augment endogenous regenerative processes and enhance functional recovery, the therapeutic effects of XSEC on post-ischemic remodeling may be augmented in combination with EE.

Magnetic resonance imaging (MRI) is a powerful means for non-invasively monitoring the structural and functional alterations of the brain (Jiang et al., 2010b). Although EE- or BYHWD-induced axonal plasticity and repair have been well-documented in histological studies, limited studies have reported using MRI to investigate the therapeutic effects of XSEC or EE monotherapy and combination therapy on axonal remodeling in cerebral ischemic rats. Accordingly, we non-invasively evaluated axonal abnormalities of ischemic brain in rats using MRI and specifically investigated the therapeutic effects of XSEC or EE monotherapy and combination therapy on axonal remodeling in cerebral ischemic rats.

Myelin is essential for axonal structure and function. Oligodendrocytes, myelin forming cells in the central nervous system (CNS), are highly vulnerable to ischemia injury (Zhang et al., 2013). Stroke acutely induces mature oligodendrocyte damage, leading to the disruption of axonal structure. Successful regeneration of oligodendrocytes is essential for remyelination after brain injuries. It has been previously demonstrated that cerebral ischemia can stimulate the proliferation of oligodendrocyte precursor cells (OPCs) in ischemic brain (Zhang et al., 2010; Franklin and Goldman, 2015). However, endogenous oligodendrogenesis in response to stroke is limited, and most of these OPCs fail to differentiate into mature oligodendrocytes to form myelin sheaths for sprouting axons following an ischemic stroke (Cafferty et al., 2008). Preclinical studies have shown that enhancement of endogenous oligodendrogenesis by cell- and pharmacological-based therapies is closely associated with the augmentation of sprouting axons in ischemic brain (Zhang et al., 2012; Ramos-Cejudo et al., 2015).

In addition to neuronal intrinsic capacity, axonal regeneration can be constrained by intrinsic myelin-associated neurite growth inhibitors. Among them, NogoA expressed by oligodendrocytes is a principal inhibitor for axonal regrowth in injured adult CNS. NogoA and its receptor NgR appear to exert inhibitory effects on regenerative axonal extension by activating intracellular components, RhoA and its downstream target Rho-associated kinase2 (ROCK2) (Pernet and Schwab, 2012; Fujita and Yamashita, 2014). Therefore, manipulations that amplify stroke-induced oligodendrogenesis and counteract neurite growth inhibitors may enhance axonal regeneration and functional recovery (Otero-Ortega et al., 2017). Thus, the present study further examined whether the combination treatment could induce endogenous oligodendrogenesis and overcome the intrinsic axonal growth-inhibitory pathways NogoA/NgR and RhoA/ROCK2, thus, providing us with valuable insight into the beneficial effects of a combined therapeutic approach of XSEC and EE interventions on axonal remodeling after an ischemic stroke.

## Materials and Methods

### Preparation of XSEC

XSEC (batch no: 20170706) was supplied by Sanmenxia Sinoway Pharmaceutical, Co., Ltd. (Henan, China). The processing of XSEC followed a strict quality control, and the main ingredients of XSEC were subjected to standardization in our previous study (Zhang et al., 2016). XSEC was dissolved in physiological saline to a concentration of 28 mg/mL before use. The dose of XSEC (140 mg/kg) used in this study was determined based on our previous study (Li et al., 2018b).

### Housing Conditions

EE housing was 90 cm long × 75 cm wide × 50 cm high, with climbing ladders, chains, different shaped tubes, plastic tunnels, and small boxes to provide sensorimotor and cognitive stimulations. Objects were changed every 2 days. Additionally, EE provided enhanced social stimulations as a total of 8–10 rats were housed together (Jiang et al., 2016). The standard housing condition was 40 cm long × 30 cm wide × 20 cm high without objects mentioned in the EE housing, and animals were housed in sets of 3 per cage.

### Animals and Experimental Design

Adult male Sprague–Dawley rats (aged 8 weeks and weighing 300–320 g) were purchased from Vital River Laboratory Animal Technology, Co., Ltd. (Beijing, China). Rats were housed in a temperature (23 ± 1°C) and humidity-controlled (55 ± 10%) environment with 12-h light–dark cycles. All experimental procedures were performed according to the National Institute of Health Guide for the Care and Use of Laboratory Animals and approved by the Capital Medical University Animal Ethics Committee (Permit number: AEEI-2018-052). Efforts were made to reduce the number of animals used and avoid their suffering wherever possible.

Focal cerebral ischemia was induced in rats by permanent occlusion of the middle cerebral artery (MCAO) as described previously (Longa et al., 1989). Briefly, the rats were anesthetized under isoflurane (5% for induction and 2% for maintenance) in N_2_O/O_2_ (70/30). The right common carotid artery (CCA), external carotid artery (ECA), and internal carotid artery (ICA) were exposed through a midline neck incision. A 4-0 monofilament nylon suture (Beijing Sunbio Biotech, Co., Ltd., China) was inserted through the right ECA and gently advanced into the lumen of the ICA to occlude the origin of the MCA. Rats showing no behavior deficits, including rotational asymmetry and dysfunctional limb placement, were excluded from the study (Ruscher et al., 2009).

Rats with successfully induced MCAO were randomly divided into four groups: (i) vehicle group, MCAO rats were treated with saline and housed in standard condition; (ii) EE group, MCAO rats were treated with saline and housed in the EE cage; (iii) XSEC group, MCAO rats were treated with XSEC and housed in standard condition; (iv) XSEC + EE group, MCAO rats were treated with XSEC and housed in the EE cage. Sham-operated rats that were subjected to the same surgical procedure but without occlusion of the MCA were grouped under the control group. Control rats were treated with saline and housed in standard condition. XSEC and XSEC + EE treated rats were intragastrically administered with XSEC once daily for 30 consecutive days starting at 24 h after MCAO. Rats in the control, vehicle, and EE groups were administered saline at 5 mL/kg in the same manner. EE and XSEC + EE treated rats were housed in EE cages at 8:00 PM each day and returned to the standard cages at 8 AM the following day for a period of 30 days after MCAO. The testing order of the animals was based on a random number list to avoid experimental bias during the tests (random was generated by: http://www.99cankao.com/numbers/random-number-generator.php).

### *In vivo* Analysis by MRI and Diffusion Tensor Imaging

MRI measurements were performed on the 31^st^ day after MCAO using a 7.0T animal MRI scanner (Bruker, PharmaScan, Germany). A total of 40 rats (8 rats per group) were used.

T2-weighted imaging (T2WI) was conducted using a fast spin-echo pulse sequence. Infarct tissues were determined from abnormal hyperintensity regions on T2 images (Chan et al., 2009). Infarct volume was calculated by multiplying the total infarct area measured on individual slices by the slice thickness (0.7 mm) (Liu et al., 2011). Similarly, hemispheric and ventricular volumes were obtained. Residual hemispheric volume was calculated by subtracting the infarct and ventricular volumes from the hemispheric volume (Plane et al., 2008). Data were expressed as the ratio of the ipsilateral residual hemispheric volume relative to the contralateral. 3D reconstruction of the infarct tissue was achieved by 3D Slicer software^1^.

Diffusion tensor imaging (DTI) was performed with an axial single-shot spin echo-planar imaging sequence (Zhang et al., 2016). The directionally encoded color (DEC), fractional anisotropy (FA), axial diffusivity (AD), and radial diffusivity (RD) maps were reconstructed by Paravision version5.1 software (Bruker, Germany). Regions of interest (ROIs) were placed in the bilateral cortex, striatum, and external capsule on DTI parametric maps according to rat atlas (Paxinos and Watson, 1986).

Next, diffusion tensor tractography (DTT) was performed using the 3D Slicer software. The mean fiber length and fiber density of the bilateral cortex, striatum, and external capsule were obtained according to our previous study (Li et al., 2018a). All data were presented as the ratio of the ipsilateral value relative to the contralateral value. All analyses were performed in a blinded manner.

### Tissue Processing

At the end of MRI scan, rats (*n* = 4 per group) were deeply anesthetized and perfused transcardially with 0.9% saline followed by 4% paraformaldehyde. The brains were removed and post-fixed in the same fixative at 4°C overnight. Brain blocks locating from −0.4 to 0.4 mm relative to the bregma were cut, processed, and embedded in paraffin. A series of 5 μm-thick coronal brain sections were sliced from the paraffin block for immunostaining. Peri-ischemic tissues of the remaining rats (*n* = 4 per group) were separated according to a previously described method (Ashwal et al., 1998) for qRT-PCR and western blot analysis to detect the expressions of axonal growth related molecules and axonal growth inhibitory molecules.

### Histological Evaluation

Luxol fast blue (LFB) staining was performed as reported previously (Shi et al., 2010). Three microscopic fields from the peri-infarct cortex, striatum, external capsule, and their contralateral homologous areas were randomly selected. Data were expressed as the ipsilateral integrated optical density (IOD) relative to the contralateral IOD.

The proliferation of OPCs was detected by double-labeled immunostaining against Ki67 (a marker of proliferating cell) and NG2 (a marker of OPC), whereas Ki67 and CNPase (a marker for oligodendrocytes) double-labeled immunostaining was performed to determine newly matured oligodendrocytes as previously described (Zou et al., 2016). Briefly, sections were stained with primary antibodies against rabbit anti-Ki67 (Abcam; ab15580; 1:100) in combination with mouse anti-NG2 (Abcam; ab50009; 1:200) or mouse anti-CNPase (Abcam; ab6319; 1:300), followed by incubation in species- and isotype-appropriate Alexa Fluor 488 and 594 secondary antibodies. For quantitative analysis, the number of Ki67^+^/NG2^+^ and Ki67^+^/CNPase^+^ cells was estimated in three randomly selected microscopic regions from the peri-infarct cortex and striatum. Data were expressed by the average number of cells per mm^2^. All images were visualized with a digital camera connected to an optical or fluorescence microscope and analyzed with NIS-Elements Basic Research Image Collection Analysis system (Nikon, Japan) by an investigator blind to the experimental groups.

### qRT-PCR Analysis

Total RNA was extracted from the peri-infarct tissues using RNAprep pure Kit (for tissue) (Tiangen, DP431, Beijing, China), and cDNA was synthetized using FastQuant RT Kit (Tiangen, KR106, Beijing, China). RT-PCR was performed on a CFX Connect^TM^ Real-Time PCR Detection System (Bio-Rad) using SuperReal PreMix Plus (Tiangen, FP205, Beijing, China). The primer sequences were as follows (forward and reverse, respectively): NG2, 5′-AGCCCATGGCC TTCACTATCAC-3′ and 5′-CCGGCCCTGAATCACTGTCTA-3′; CNPase, 5′-GGAGACATAGTGCCCGCAAAG-3′ and 5′-TTGCACTCGTGCAGCGTA-3′; growth associated protein-43 (GAP-43), 5′-CACCATGCTGTGCTGTATGAGAA-3′ and 5′ -GTCCACGGAAGCTAGCCTGA-3′; NogoA, 5′-CTTGGTCA TGTGAACAGCACAATAA-3′ and 5′-CATTGAACAAGGCAC CAACGTAA-3′; NgR, 5′-TCCAGTCATGCCGAAATCTCAC-3′ and 5′-TGGTAGGGTCCACGACATGAAG-3′; RhoA, 5′-CA GCAAGGACCAGTTCCCAGA-3′ and 5′-AGCTGTGTCCCAT AAAGCCAACTC-3′; ROCK2, 5′-CTAACAGTCCGTGGGTGG TTCA-3′ and 5′-CTCAGGCACATCATAATTGCTCATC-3′; β-actin, 5′-GGAGATTACTGCCCTGGCTCCTA-3′ and 5′-GACTCATCGTACTCCTGCTTGCTG-3′. The reactive conditions were as follows: denaturing at 95°C for 15 min, followed by 40 cycles of 95°C for 10 s, 55°C for 31 s, and 72°C for 30 s. Data were presented as relative mRNA level according to the 2^–ΔΔ*Ct*^ method. Actin served as an internal standard.

### Western Blot Analysis

Peri-ischemic tissues were homogenized in radioimmunoprecipitation assay (RIPA) buffer (Applygen, Beijing, China). Protein concentration was determined, and equal amounts of proteins (21 μg per lane) were separated on 10% sodium dodecyl sulfate (SDS)-polyacrylamide gels, and then transferred onto polyvinylidene difluoride membranes (Millipore, Billerica, MA, United States). Membranes were blocked with 5% non-fat milk for 1 h at room temperature and then incubated overnight at 4°C with the primary antibodies against the following proteins: NG2 (1:2000), CNPase (1:2000), GAP-43 (Epitomics, #2259-1, 1:5000), NogoA (Abcam; ab62024; 1:5000), NgR (Abcam; ab26291; 1:5000), RhoA (Abcam; ab68826; 1:20000), ROCK2 (Abcam; ab125025; 1:40000), and GAPDH (Neobioscience; NBL01c; 1:40000). After washing, the membranes were incubated with secondary horseradish peroxidase-labeled anti-rabbit (CWBIO; CW0103S; 1:20000) or anti-mouse (CWBIO; CW0102S; 1:20000) IgG. Immunoreactive bands were visualized using an enhanced chemiluminescent kit (Aspen Biotechnology, Hubei, China). The optical densities of the protein bands were measured using the ImageJ software. GAPDH served as a loading control.

### Behavioral Study

A total of 50 rats (9–11 rats per group) were submitted to spatial learning and memory test in the Morris water maze (MWM) without undergoing MRI and histological studies at days 31–36 after MCAO (Jing et al., 2015). The reason was that a previous MRI study had demonstrated that rats who were subjected to short periods of training in the MWM show structural modifications and rapid changes in diffusion MRI indices (Blumenfeld-Katzir et al., 2011; Hofstetter and Assaf, 2017). Moreover, behavioral training and testing may increase the number of hippocampal neurons (Gould et al., 1999) and change the regional gray matter volume (Sumiyoshi et al., 2014).

The MWM consisted of a circular tank with a circular transparent platform (15 cm in diameter). The tank was filled with water (18–20°C) and divided into four virtual quadrants (quadrants I, II, III, and IV). During spatial acquisition test, the platform was placed 1.5 cm below the water surface in the middle of quadrant I to serve as the goal. The animals received four trials a day for four consecutive days. For each trial, the rat was allowed to swim for 60 s to find the submerged platform. If the rat failed to locate the platform within 60 s, it was guided to the platform and left on it for 10 s. The path length taken by the animals to locate the platform was recorded and analyzed with a video tracking system (JLBehv-MWMG, Jiliang Software Technology, Co., Ltd., Shanghai). To assess spatial memory retention, the probe trial was performed 24 h after the last acquisition trial. The platform was removed from the maze, and the rats were allowed to swim for 30 s. The percentage of distance traveled by the rats in the trained quadrant (quadrant I) was calculated.

Following the probe trial, the animals were subjected to a platform-switched learning task to assess repeated reversal spatial learning and memory (Morris et al., 1982). Briefly, the hidden platform was placed in the center of quadrant II for the first trial, and moved to quadrant III or quadrant IV on the second and third trial, respectively. For each trial, the rats had 60 s to locate the hidden platform. The swimming track of rats to the new location was recorded, and the path length taken by the animals to locate the platform was analyzed. An experimenter blind to the group information performed data analysis.

### Statistical Analysis

All data were expressed as mean ± standard error of the mean (SEM). Statistical analyses were performed using the SPSS 21.0 (SPSS, Inc., United States) software. Data from the MWM acquisition test were analyzed using two-way repeated measures ANOVA (between subject factor–treatment; within subject factor-time) with Bonferroni’s *post hoc* test. Data from the probe trial, platform-switched test, MRI, and histological measurements were analyzed by one-way ANOVA, followed by Bonferroni’s *post hoc* test. Significance was defined as *P* < 0.05.

## Results

### Combination of XSEC and EE Therapy Reduced Infarct Volume and Alleviated Tissue Atrophy in MCAO Rats

**FIGURE 1:**
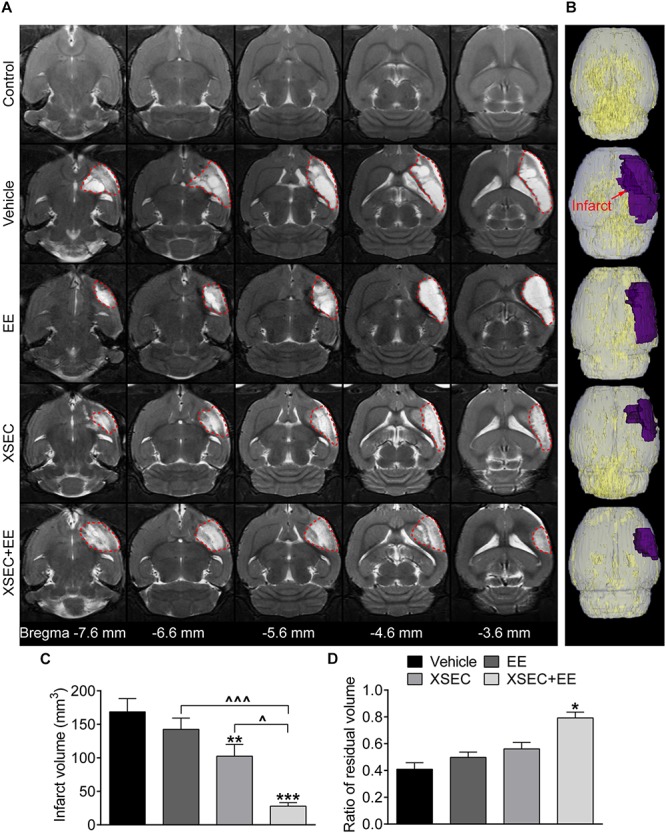
Effect of XSEC or EE and their combination on the infarct volume in MCAO rats. **(A)** Representative axial T2 images (bregma –7.6 to –3.6 mm) obtained from various group rats on the 31^st^ day after MCAO. The infarct tissues were surrounded by red dotted lines. **(B)** Typical 3D reconstruction images of ischemic lesion were acquired by 3D Slicer software (purple region indicated the infarction). **(C,D)** Quantitative analysis of the infarct volume and the ratio of residual volume, respectively (one-way ANOVA followed by Bonferroni’s *post hoc* test, *n* = 8 per group). ^∗^*P* < 0.05, ^∗∗^*P* < 0.01, ^∗∗∗^*P* < 0.001 vs. vehicle group. ^∧^*P* < 0.05, ^∧∧∧^*P* < 0.001 vs. XSEC + EE group.

T2WI was conducted to measure the infarct size on the 31^st^ day after MCAO. Axial T2 images exhibited normal structure in the control rats. Abnormal hyperintense signals indicating tissue infarction were primarily detected in the ipsilateral cortex, striatum, and external capsule after MCAO (Figures 1A,B). Treating rats with XSEC + EE combination or XSEC alone significantly reduced the infarct volume compared with that in vehicle group (*P* < 0.001 or *P* < 0.01, Figure 1C). Notably, XSEC + EE combination produced a remarkable decrease in the infarct volume compared to XSEC (*P* < 0.05) or EE (*P* < 0.001) alone.

To determine chronic cerebral atrophy after MCAO, residual volume of the ipsilateral hemisphere was measured. When compared with the vehicle group, XSEC + EE robustly elevated the ratio of residual volume (*P* < 0.05), while XSEC or EE monotherapy showed no statistic differences on the ratio of residual volume (Vehicle, 0.4094 ± 0.0482; XSEC, 0.5615 ± 0.0478; EE, 0.4983 ± 0.0386; XSEC + EE, 0.7922 ± 0.0436, Figure 1D), indicating that the combined actions of XSEC and EE significantly alleviated atrophy of the ipsilateral hemisphere. Notably, XSEC + EE exhibited a slight increase in the ratio of residual volume compared to the XSEC or EE monotherapy groups, however, no statistical difference was detected.

### Combination of XSEC and EE Therapy Alleviated Axonal Microstructural Damage in MCAO Rats Based on MRI Parameters

**FIGURE 2:**
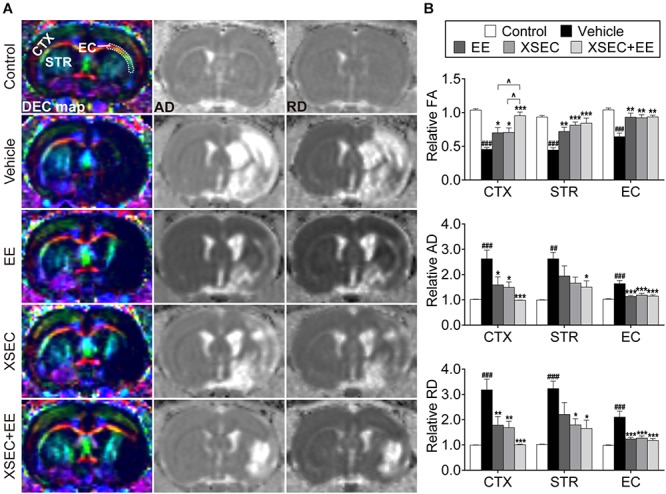
Effect of XSEC or EE and their combination on the axonal microstructural changes in MCAO rats. **(A)** Representative DEC, AD, RD maps (bregma –0.6 mm) were obtained from various group rats on the 31^st^ day after MCAO. ROIs of the cortex (CTX), striatum (STR), and external capsule (EC) were identified on the DEC map. **(B)** Quantitative analysis of the relative FA, AD, and RD in the ipsilateral cortex, striatum and external capsule (one-way ANOVA followed by Bonferroni’s *post hoc* test, *n* = 8 per group). ^##^*P* < 0.01, ^###^*P* < 0.001 vs. control group. ^∗^*P* < 0.05, ^∗∗^*P* < 0.01, ^∗∗∗^*P* < 0.001 vs. vehicle group. ^∧^*P* < 0.05 vs. XSEC + EE group.

DTI was performed to examine microstructural changes in the axons on the 31^st^ day after MCAO (Figure 2A). Significantly decreased relative FA but increased relative AD and RD were observed in the ipsilateral cortex, striatum, and external capsule of the vehicle rats compared with those in the control rats (*P* < 0.01 or *P* < 0.001, Figure 2B). XSEC and EE monotherapies and XSEC + EE combination profoundly increased the relative FA in the corresponding regions and decreased the relative AD and RD in the ipsilateral cortex and external capsule compared with those in the vehicle group (*P* < 0.05–0.001). In addition, rats treated with XSEC alone or XSEC + EE combination exhibited alleviated relative RD in the ipsilateral striatum compared with those in the vehicle rats (*P* < 0.05). In particular, increased relative FA in the ipsilateral cortex was observed with XSEC + EE combination compared to that with XSEC or EE monotherapy (*P* < 0.05). Notably, only XSEC + EE combination decreased the relative AD in the ipsilateral striatum compared with that in the vehicle group (*P* < 0.05).

### Combination of XSEC and EE Therapy Facilitated Axonal Restoration in MCAO Rats

**FIGURE 3:**
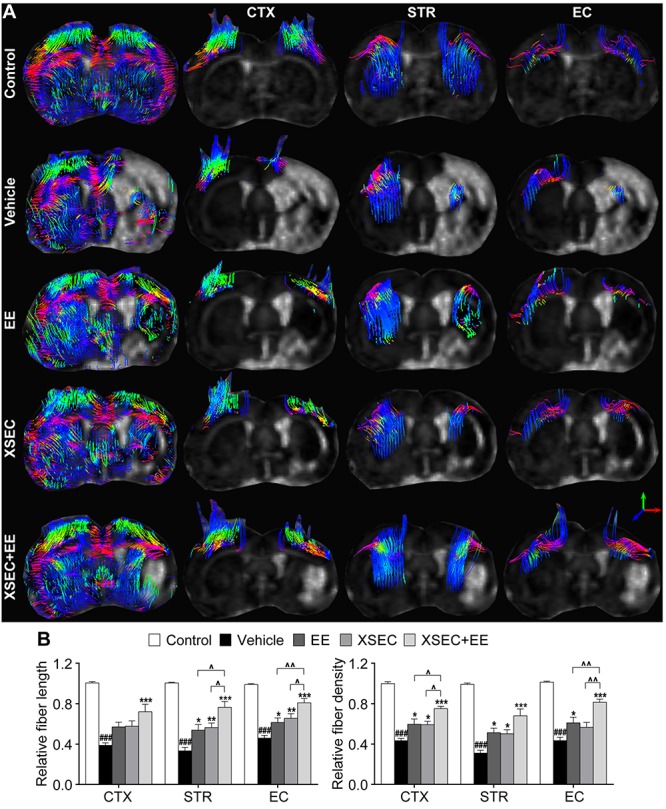
Effect of XSEC or EE and their combination on the axonal restoration in MCAO rats. **(A)** Diffusion tensor tractography (DTT) of the brain at bregma –0.6 mm and the anatomical pathway of the cortex (CTX), striatum (STR), and external capsule (EC), respectively. **(B)** Quantitation of the relative fiber length and fiber density in the ipsilateral cortex, striatum and external capsule (one-way ANOVA followed by Bonferroni’s *post hoc* test, *n* = 8 per group). ^###^*P* < 0.001 vs. control group. ^∗^*P* < 0.05, ^∗∗^*P* < 0.01, ^∗∗∗^*P* < 0.001 vs. vehicle group. ^∧^*P* < 0.05, ^∧∧^*P* < 0.01 vs. XSEC + EE group.

Diffusion tensor tractography was performed to detect the integrity and connectivity of the nerve fibers (Figure 3A). Quantitative data showed that the relative fiber length and fiber density reduced dramatically in the ipsilateral cortex, striatum, and external capsule of the vehicle rats in comparison with those in the control rats (*P* < 0.001, Figure 3B). XSEC or EE monotherapy and combination therapy significantly elevated the relative fiber length in the ipsilateral striatum and external capsule and increased the relative fiber density in the ipsilateral cortex and striatum compared with that in the vehicle group (*P* < 0.05–0.001). Furthermore, rats treated with XSEC + EE combination and EE monotherapy also exhibited increased relative fiber density in the ipsilateral external capsule compared with that in the vehicle rats (*P* < 0.05). In particular, XSEC + EE combination further resulted in higher fiber length in the ipsilateral striatum and external capsule and higher fiber density in the cortex and external capsule compared to that by XSEC or EE monotherapy (*P* < 0.05–0.01). Of note, only XSEC + EE combination elevated the relative fiber length in the ipsilateral cortex compared with that in the vehicle group (*P* < 0.001).

### Combination of XSEC and EE Therapy Preserved Myelinated Axons in MCAO Rats

**FIGURE 4:**
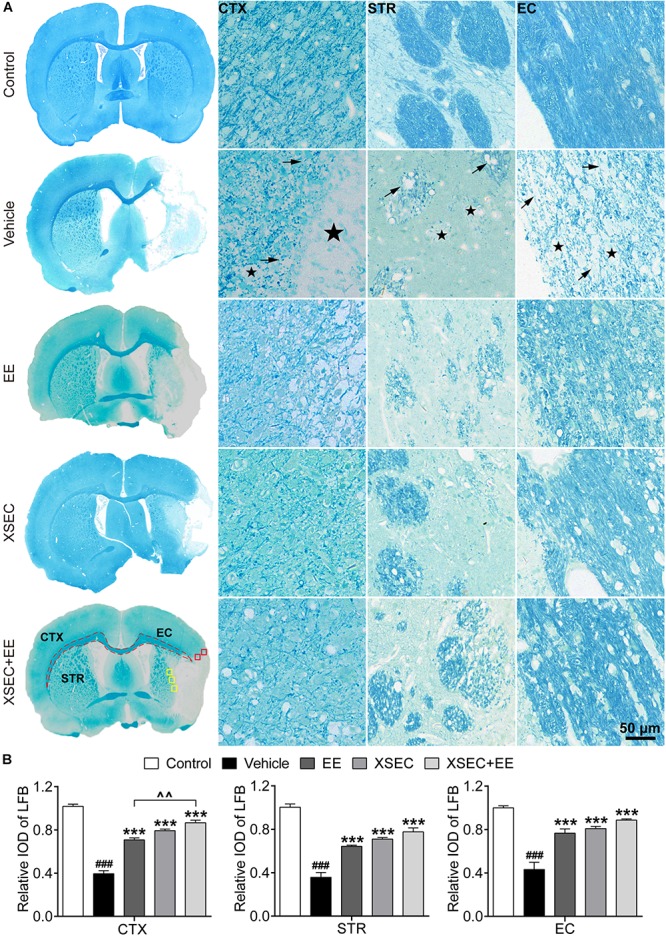
Effect of XSEC or EE and their combination on the myelinated axons in MCAO rats. **(A)** Typical LFB staining photographs showing the alterations of myelin sheath in the ipsilateral cortex, striatum and external capsule. ROIs of the peri-infarct cortex (CTX, red boxes), striatum (STR, yellow boxes), and external capsule (EC, dashed line) were indicated in the representative LFB map from a XSEC + EE treated rat. Remarkable cavitation areas (stars) and partial loss of myelin sheaths (arrows) in the axonal tracts were observed. **(B)** Quantitative data of the relative IOD of LFB in the ipsilateral cortex, striatum and external capsule (one-way ANOVA followed by Bonferroni’s *post hoc* test, *n* = 4 per group). ^###^*P* < 0.001 vs. control group. ^∗∗∗^*P* < 0.001 vs. vehicle group. ^∧∧^*P* < 0.01 vs. XSEC + EE group.

Luxol fast blue staining was performed to detect myelinated axons (Figure 4A). For the vehicle rats, LFB staining showed marked cavitation areas and myelin loss in the axonal tracts in the ischemic cortex, striatum, and external capsule, suggesting that ischemia directly injures axons and the myelin structure. However, XSEC or EE monotherapy and combination markedly increased the relative IOD of LFB in the peri-infarct cortex, striatum, and external capsule compared with that in the vehicle group (*P* < 0.001, Figure 4B). It is noteworthy that XSEC + EE combination produced a significantly higher relative IOD of LFB in the peri-infarct cortex than that by EE monotherapy (*P* < 0.01).

### Combination of XSEC and EE Therapy Enhanced OPCs Proliferation and Maturation in MCAO Rats

**FIGURE 5:**
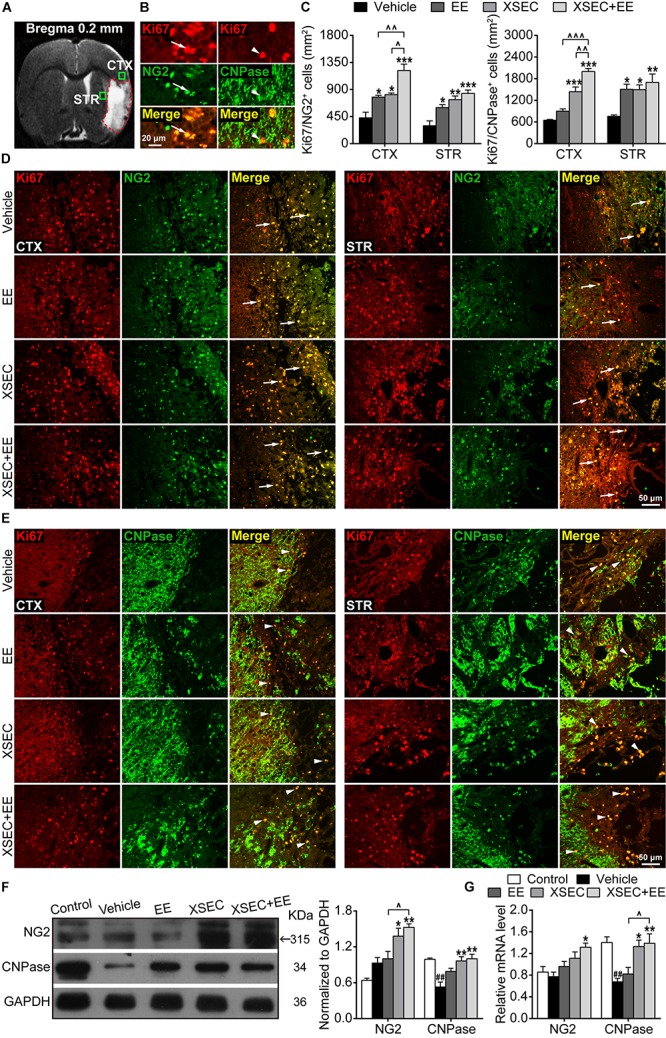
Effect of XSEC or EE and their combination on oligodendrogenesis in MCAO rats. **(A)** A representative coronal T2WI image at the level of bregma 0.2 mm. Boxes indicated the peri-infarct cortex and striatum where images were taken. **(B)** Representative images showing the co-expressed cells of Ki67^+^/NG2^+^ and Ki67^+^/CNPase^+^. **(C)** Quantitative data of the number of Ki67^+^/NG2^+^ and Ki67^+^/CNPase^+^ co-expressed cells in the peri-infarct cortex and striatum. **(D,E)** Representative images of Ki67/NG2 and Ki67/CNPase double immunofluorescent staining in the peri-infarct cortex and striatum. **(F,G)** Quantitative analysis of the protein and mRNA level of NG2 and CNPase in the peri-infarct brain tissues, respectively (one-way ANOVA followed by Bonferroni’s *post hoc* test, *n* = 4 per group). ^##^*P* < 0.01 vs. control group. ^∗^*P* < 0.05, ^∗∗^*P* < 0.01, ^∗∗∗^*P* < 0.001 vs. vehicle group. ^∧^*P* < 0.05, ^∧∧^*P* < 0.01, ^∧∧∧^*P* < 0.001 vs. XSEC + EE group.

The proliferation of OPCs was detected by double-labeled immunostaining against Ki67 and NG2 (Figures 5A,B,D). XSEC and EE monotherapies and XSEC + EE combination all significantly increased the number of Ki67^+^/NG2^+^ cells in the peri-infarct cortex and striatum compared with those in the vehicle group (*P* < 0.05–*P* < 0.001, Figure 5C). Notably, combination treatment produced a considerably higher number of Ki67^+^/NG2^+^ cells in the peri-infarct cortex compared with that by XSEC or EE monotherapy (*P* < 0.05 or *P* < 0.01), suggesting that XSEC + EE combination facilitated OPCs proliferation.

Further, Ki67 and CNPase double-labeled immunostaining was performed to determine the maturation of OPCs (Figures 5A,B,E). Compared to the vehicle group, XSEC + EE combination and XSEC monotherapy both markedly increased the number of Ki67^+^/CNPase^+^ cells in the peri-infarct cortex and striatum (*P* < 0.05–0.001, Figure 5C), and a higher number of Ki67^+^/CNPase^+^ cells was detected in the peri-infarct striatum of EE-treated rats (*P* < 0.05). Notably, XSEC + EE combination enhanced the number of Ki67^+^/CNPase^+^ cells in the peri-infarct cortex, which was significantly higher compared to that by XSEC (*P* < 0.01) or EE monotherapy (*P* < 0.05). This result suggested that the XSEC + EE combination enhanced the generation of new myelinating oligodendrocytes.

Moreover, protein and mRNA levels of NG2 and CNPase were analyzed by western blot and qRT-PCR. XSEC + EE combination and XSEC monotherapy significantly elevated the mRNA level of CNPase and protein expression of NG2/CNPase compared with that in the vehicle group (*P* < 0.05 or *P* < 0.01, Figures 5F,G). Notably, only XSEC + EE combination increased NG2 mRNA level compared with that in the vehicle rats (*P* < 0.05). In addition, the combination treatment resulted in significantly upregulated protein expression of NG2 and mRNA level of CNPase compared with those by EE monotherapy (*P* < 0.05).

### Combination of XSEC and EE Therapy Elevated Axonal Growth Marker and Suppressed Axonal Growth Inhibitors in MCAO Rats

**FIGURE 6:**
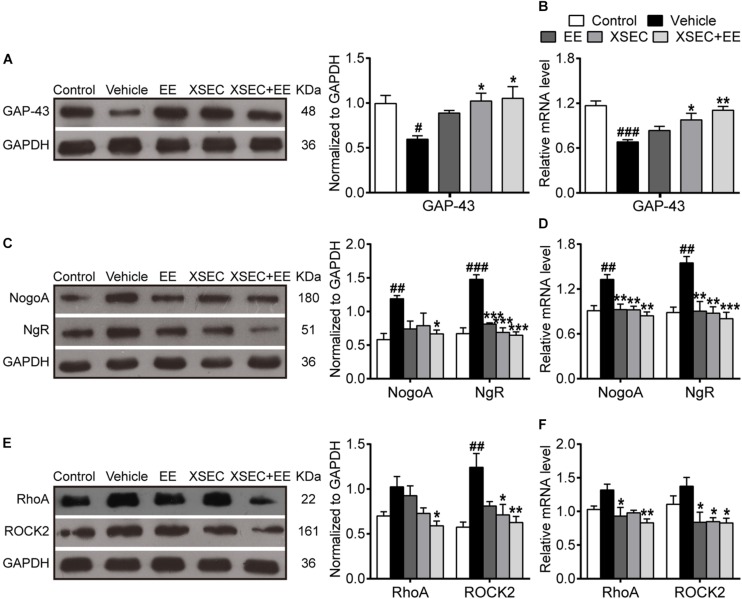
Effect of XSEC or EE and their combination on the expressions of GAP-43, NogoA/NgR and RhoA/ROCK2 in MCAO rats. Quantitative analysis of the protein **(A,C,E)** and mRNA **(B,D,F)** levels of GAP-43, NogoA/NgR, and RhoA/ROCK2, respectively (one-way ANOVA followed by Bonferroni’s *post hoc* test, *n* = 4 per group). ^#^*P* < 0.05, ^##^*P* < 0.01, ^###^*P* < 0.001 vs. control group. ^∗^*P* < 0.05, ^∗∗^*P* < 0.01, ^∗∗∗^*P* < 0.001 vs. vehicle group.

GAP-43, a marker for axonal growth cones, was examined to evaluate axonal outgrowth (Sandelius et al., 2018). A significant decrease in the mRNA and protein expression of GAP-43 was observed in the vehicle rats compared with those in the control rats (*P* < 0.05–0.001, Figures 6A,B). However, XSEC monotherapy and XSEC + EE combination significantly upregulated GAP-43 mRNA and protein expression compared with those in the vehicle group (*P* < 0.05 or *P* < 0.01).

As axonal outgrowth could be hindered by intrinsic axonal growth inhibitory molecules, the expressions of NogoA, NgR, RhoA, and ROCK2 were measured in the peri-infarct tissue. Compared to the control group, the expressions of NogoA/NgR protein and mRNA increased noticeably in the vehicle group (*P* < 0.01 or *P* < 0.001, Figures 6C,D). XSEC and EE monotherapies and XSEC + EE combination significantly downregulated NgR protein and NogoA/NgR mRNA expressions compared with those in the vehicle group (*P* < 0.01 or *P* < 0.001). Notably, XSEC + EE combination further attenuated NogoA protein expression compared with that in the vehicle group (*P* < 0.05). Moreover, mRNA and protein levels of RhoA and ROCK2 were significantly downregulated by the combination treatment compared with that in the vehicle group (*P* < 0.05 or *P* < 0.01, Figures 6E,F). Moreover, rats treated with XSEC monotherapy showed reduced ROCK2 protein and mRNA levels, whereas EE-treated rats exhibited alleviated mRNA level of RhoA/ROCK2 compared with that in the vehicle rats (*P* < 0.05).

### Combination of XSEC and EE Therapy Improved Cognitive Function in MCAO Rats

**FIGURE 7:**
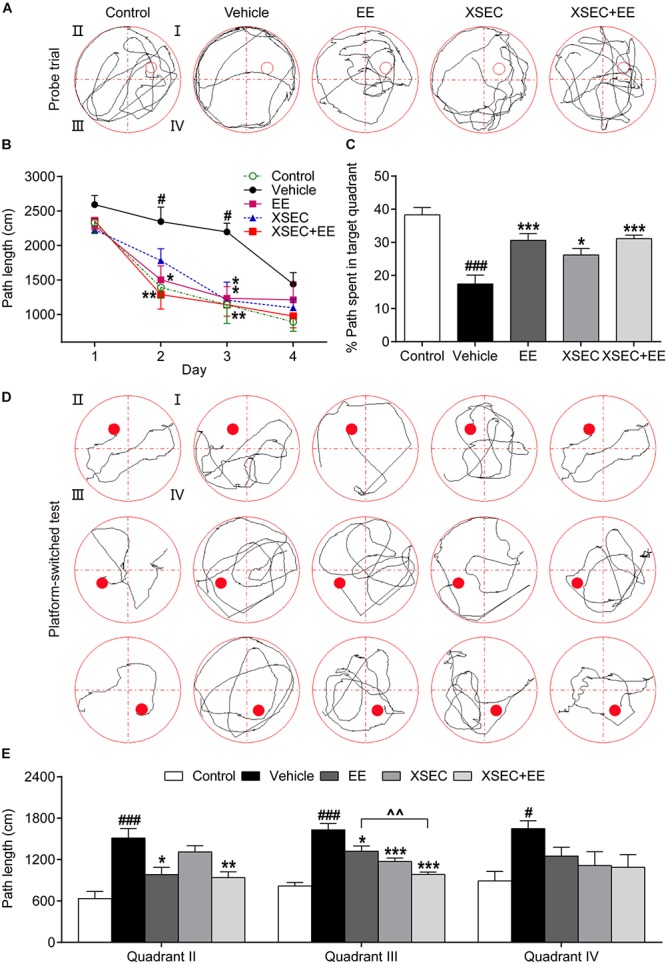
Effect of XSEC or EE and their combination on spatial learning and memory in MCAO rats. **(A)** Swimming traces of the rats in the probe trial. **(B)** The path length taken by rats to find the submerged platform in the spatial acquisition trial (two-way ANOVA with repeated measures, *n* = 9–11 per group). **(C)** The percentage of swimming distances of rats spent in the target quadrant (quadrant I) during the probe trial. **(D)** Swimming traces of the rats in the platform-switched test. **(E)** The path length of the rats traveled to locate the platform in the platform-switched test (one-way ANOVA followed by Bonferroni’s *post hoc* test for **C,E**, *n* = 9–11 per group). ^#^*P* < 0.05, ^###^*P* < 0.001 vs. control group. ^∗^*P* < 0.05, ^∗∗^*P* < 0.01, ^∗∗∗^*P* < 0.001 vs. vehicle group. ^∧∧^*P* < 0.01 vs. XSEC + EE group.

The MWM task was performed to assess the spatial learning and memory capacity of all rats. In the spatial acquisition test, repeated measures of ANOVA showed significant overall effects of time and treatment on the path length (time, *F*_(__3_,_13__5__)_ = 46.098, *P* < 0.001; treatment, *F*_(__4_,_45__)_ = 7.847, *P* < 0.001). These results indicated improved spatial learning over time in all testing rats, and the treated rats showed better learning acquisition than the vehicle rats. *Post hoc* analysis revealed that vehicle rats covered longer swimming distances to find the submerged platform on the 2^nd^ and 3^rd^ training day compared with that by the control rats (*P* < 0.05, Figure 7B), indicating impaired spatial learning after MCAO. Treatment with either EE monotherapy or XSEC + EE combination significantly reduced swimming distances by rats to locate the hidden platform on the 2^nd^ and 3^rd^ training day (*P* < 0.05*–*0.01). Moreover, XSEC-treated rats also traveled shorter swimming distances than vehicle rats on the 3^rd^ training day (*P* < 0.05).

In the probe trial (Figure 7A), vehicle rats exhibited lower percentages of distance traveled in the target quadrant compared with that by the control rats (*P* < 0.001, Figure 7C), demonstrating that the memory retention capacity was reduced after MCAO. XSEC-, EE-, and XSEC + EE-treated rats displayed better memory retention, as evidenced by the significantly higher percentage of distance traveled in the target quadrant than the vehicle rats (*P* < 0.05 or *P* < 0.001).

In the platform-switched learning trial (Figure 7D), vehicle rats showed much longer swimming distances to find the location of the changed platform than those by the control rats (*P* < 0.05 or *P* < 0.001, Figure 7E). EE monotherapy and XSEC + EE combination rats exhibited a clear preference for the new target quadrant with much shorter swimming distances to locate the switched platform in quadrants II and III compared to those by the vehicle rats (*P* < 0.05–0.001). XSEC-treated rats also showed shorter distances to find the platform in quadrant III compared to those by the vehicle rats (*P* < 0.001). Of note, rats treated with XSEC + EE combination traveled significantly shorter distances to find the platform than the EE-treated rats in quadrant III (*P* < 0.01).

## Discussion

Although several lines of evidence have demonstrated that XSEC and EE may independently promote brain plasticity and neurological recovery after an ischemic stroke (Nygren et al., 2006; Chen et al., 2015, 2017; Zhang et al., 2016), the efficacy of a combination of XSEC and EE on post-stroke axonal injury and remodeling still needed to be elucidated. Based on our MRI and histological methodologies, the present study demonstrated that the combined actions of XSEC and EE exerted synergistic effects on reducing cerebral atrophy, facilitating axonal remodeling, and improving spatial learning and memory in a permanent MCAO rat model accompanied with amplifying stroke-induced oligodendrogenesis and overcoming the intrinsic axonal growth inhibitory signals, which was beneficial to axonal outgrowth.

Magnetic resonance imaging can non-invasively monitor neuronal remodeling related to neurological outcome after brain injury (Jiang et al., 2010a). T2WI is a favorable tool to determine anatomical characteristics and pathological changes of the brain (Liu et al., 2011). In the present study, T2WI images showed that a large scale of infarction was located in the ipsilateral MCA territory in MCAO rats. XSEC individually decreased the infarct volume compared with that in vehicle group, and this effect was further boosted by the combined application of XSEC and EE. Notably, EE monotherapy did not produce beneficial effects in restoring brain infarct volume, which was in agreement with previous studies (Soderstrom et al., 2009; Xie et al., 2019). It should be also be noted that the combination treatment had an added beneficial effect as evidenced by significantly reduced atrophy of the ipsilateral hemisphere following an ischemic stroke, whereas the monotherapies showed no effect. This finding suggested that the combination treatment might provide additive benefits for reducing brain tissue loss in MCAO rats.

Axonal sprouting and regeneration are critical processes during brain repair after stroke injury and are related to improvements in neurologic deficits after a stroke (Ueno et al., 2012). DTI has been widely applied to non-invasively monitor microstructural alterations of the white matter and fiber-rich gray matter in animals and patients with ischemic stroke (Bai et al., 2015; Jung et al., 2017). Using this technique, we assessed the effects of XSEC and EE separately or in combination on axonal recovery. The most commonly applied parameter from DTI is FA. FA characterizes the density, distribution, and orientational coherence of the axons (Granziera et al., 2007). The other DTI-derived parameters AD and RD may be useful to elucidate the changes in axons and myelin, respectively (Aung et al., 2013). In the present study, decreased FA accompanied with increased AD and RD were identified in the cortex, striatum, and external capsule of the ischemic hemisphere in the vehicle rats. In general, a decrease in FA in the lesioned area represents demyelination and axonal degeneration (Tuor et al., 2014). Meanwhile, the phenomenon of increased AD has been attributed to structural breakdown of axons, whereas enhanced RD may result from widespread myelin degradation after stroke (Pitkonen et al., 2012). DTI data in this study indicated widespread loss of integrity of axons and myelin in a rat model of 30-days permanent MCAO. Notably, XSEC + EE combination displayed increased FA accompanied by reduced AD and RD in the cortex, striatum, and external capsule ipsilateral to the site of ischemic injury. In particular, FA of the combination treatment group remained significantly higher in the ipsilateral cortex compared to that in XSEC or EE monotherapy. The increased FA is positively associated with the reorganization of white matter, e.g., due to axonal remodeling (Wiesmann et al., 2017). The attenuation in AD and RD corresponds with axonal growth and myelination, respectively (Jiang et al., 2010a). Thus, DTI parameters presented here suggested that the combined intervention alleviated white matter lesions and facilitated axonal reorganization following an ischemic stroke. In correspondence with DTI results, DTT maps revealed that decreased axonal projections emanated from the ischemic striatum, cortex, and external capsule in the vehicle rats. However, XSEC + EE combination robustly elevated fiber density in the ischemic cortex and external capsule and increased fiber length in the striatum and external capsule compared to those with the monotherapies, suggesting an improvement in the fiber tract repair. These results were in agreement with the morphological study of myelin fibers by LFB staining. Histological results showed that ischemic rats treated with XSEC and EE combination had a significantly higher density of LFB positive axons in the perilesional brain regions compared with that in the vehicle rats. Based on the results of MRI and histopathological analysis, the present study demonstrated the better effect of XSEC + EE combination therapy on facilitating axonal remodeling. These findings highlighted the therapeutic potential of the combined action of XSEC and EE for long-term rehabilitation after an ischemic stroke.

Oligodendrogenesis plays a critical role in axonal reorganization after stroke. Although the repair capacity of the CNS is limited, regeneration of mature oligodendrocytes has been observed in the peri-infarct areas after stroke (Zhang et al., 2010; Franklin and Goldman, 2015). In this study, we found that MCAO rats exhibited an increase in the numbers of NG2-positive OPCs and newly generated CNPase-positive oligodendrocytes along the peri-infarct striatum and cortex, whereas they were not detected in the undamaged hemisphere of MCAO rats and intact brain of the control rats. Our results were consistent with previous reports indicating that a stroke increased oligodendrogenesis in the peri-infarct areas (Zhang et al., 2010; Ramos-Cejudo et al., 2015). Although far from being completely understood, accumulating evidences have suggested that a complex interaction of extrinsic and intrinsic cues from the ischemic microenvironment would impact the proliferation and differentiation of OPCs after a stroke (Moore et al., 2011; Zhang et al., 2013). Experimental evidences have indicated that astrocyte-secreted factors could positively or negatively influence OPCs proliferation, differentiation, and survival during CNS injury and demyelination (Moore et al., 2011). For example, insulin-like growth factor-1 (IGF-1) secreted by astrocytes supports the proliferation of oligodendrocytes and generation of myelinating oligodendrocytes under conditions of brain injury (Chesik et al., 2008). Bone morphogenic proteins (BMPs) expressed by reactive astrocytes during adult CNS injury block the process of oligodendrocyte differentiation (See and Grinspan, 2009). Moreover, the M1-macrophages of microglial cells, dominating early after demyelination, support the proliferation and migration of OPCs, whereas the M2-dominant phenotype in demyelinated lesions is essential for efficient remyelination by promoting oligodendrocyte differentiation (Miron et al., 2013; Braun et al., 2016).

It should be noted that endogenous oligodendrogenesis in response to a stroke is limited. Although a stroke could induce the proliferation of OPCs, most of these OPCs fail to develop into mature oligodendrocytes, resulting in insufficient remyelination (Zhang et al., 2013). Therefore, understanding the molecular environment of injured cerebral tissue will help to understand endogenous oligodendrogenesis in response to stroke and thus, help in designing therapeutic approaches for the amplification of these cells, which may lead to efficient remyelination and axonal regeneration.

EE enhances newborn glial scar astroglia and NG2-positive OPCs in the post-ischemic neocortex, which may be beneficial for brain repair and post-stroke plasticity (Komitova et al., 2006). In parallel, the present study showed that XSEC and EE separately or in combination enhanced OPCs proliferation and increased the numbers of newly generated mature oligodendrocytes in the vicinity of the ischemic regions compared with that in the vehicle rats. Of note, XSEC + EE combination resulted in higher numbers of NG2-positive OPCs and CNPase-positive oligodendrocytes in the peri-infarct cortex compared with that by XSEC or EE monotherapy. Furthermore, the protein and mRNA levels of NG2 and CNPase increased significantly by XSEC + EE combination treatment. These results, along with the information obtained from DTI, DTT, and histopathological presentations, likely reflected that the combined application of XSEC and EE enhanced the production of OPCs and increased the number of newly generated oligodendrocytes, leading to substantial remyelination and increased myelinated axons in the peri-infarct regions. These may be important mechanisms for the beneficial effect of XSEC + EE combination.

Despite the requirement of intrinsic factors from regenerating axons, myelin-associated neurite growth inhibitors, as a potential mechanism, limit axonal regeneration and functional recovery after an ischemic stroke (Schwab and Strittmatter, 2014). Several studies have indicated that some specific neurite growth inhibitory factors enriched in myelin could limit axonal regeneration and functional recovery after an ischemic stroke (Kilic et al., 2010; Fujita and Yamashita, 2014). Among them, NogoA expressed by oligodendrocytes is a principal inhibitor for axonal regrowth in the injured CNS. NogoA binding to the NgR complex activates RhoA and its downstream target ROCK2, ultimately leading to axonal growth cone collapse and neurite outgrowth inhibition (Etienne-Manneville and Hall, 2002; Kilic et al., 2010; Fujita and Yamashita, 2014; Schwab and Strittmatter, 2014). Hence, strategies that aim at the inhibition of NogoA signaling can promote axonal regeneration and behavioral recovery (Pernet and Schwab, 2012). The present study demonstrated that XSEC + EE combination significantly lessened cerebral atrophy and induced axonal reorganization in perilesional brain regions. In light of this, we further explored whether the separate and combined actions of XSEC and EE could overcome the intrinsic axonal growth inhibitory pathways to facilitate axonal remodeling. According to the results of this study, XSEC or EE monotherapy was sufficient to counteract ischemic stroke-induced high expression of NgR protein and NogoA/NgR/ROCK2 mRNA. In particular, XSEC + EE combination markedly downregulated the protein expressions of NogoA and RhoA compared to that in the vehicle group. To further confirm that NogoA/NgR and RhoA/ROCK2 downregulation by XSEC and EE combination could promote axonal elongation, we assessed the axonal growth marker GAP-43, which was primarily localized in the axonal growth cone. Our current result revealed significantly reduced protein and mRNA levels of GAP-43 in vehicle-treated MCAO rats, whereas XSEC + EE combination remarkably enhanced protein and mRNA levels of GAP-43 compared to those in the vehicle group. The high level of GAP-43 is associated with axonal extension and cytoskeletal reorganization (Loncarevic-Vasiljkovic et al., 2009). Based on this, our data strongly suggested that XSEC + EE combination could enhance axonal regrowth by overcoming intrinsic growth inhibitory signals.

Axonal damage leads to persistent deficits with limited recovery of function (Kilic et al., 2010; Fujita and Yamashita, 2014). Promoting axonal reorganization is a primary contributor in cognitive function recovery during brain repair after stroke injury (Zatorre et al., 2012). In light of this, we investigated whether the enhancement in axonal remodeling by XSEC + EE combination might contribute to the improvement in learning and memory performance in the MWM task. Our results were consistent with previous reports and indicated that XSEC and EE monotherapies improved spatial reference learning and memory performance, as evaluated by the MWM test (Dahlqvist et al., 2004; Li et al., 2018b). Interestingly, the combined action of both permitted faster adaptation to a changed testing environment, as revealed in the platform-switched learning trial. In this task, rats must eliminate their initial learning and learn new information to find the repeatedly changing platform (Vorhees and Williams, 2006). Previous studies have supported that cortical and striatal dysfunction may be closely linked to specific reversal learning impairments (Kesner and Churchwell, 2011; Wei et al., 2011). With the information obtained from DTI analysis and histological evaluation, we demonstrated that XSEC + EE combination induced axonal reorganization and amplified oligodendrogenesis around peri-infarct tissues. These findings raised the interesting possibility that the faster adaptation to a changed testing environment by XSEC + EE combination treatment may be related to axonal remodeling in the peri-infarct regions.

There are several limitations in our study that should be mentioned. Firstly, the main anti-ischemic stroke components of XSEC needed to be further investigated. Based on the traditional documentation of BYHWD, XSEC is prepared from seven medicinal herbs, viz., *Astragalus membranaceus*, *Angelica sinensis*, *Paeonia lactiflora*, *Ligusticum chuanxiong*, *Prunus persica*, *Carthamus tinctorius*, and *Pheretima aspergillum*. All the herbs are recorded in the Chinese Pharmacopoeia. *In vivo* and *in vitro* experiments have confirmed that some of the active constituents isolated from these crude herbs have beneficial effects on preventing damage to the brain. *A. membranaceus* is the major component of XSEC. Many active compounds, such as astragaloside IV and calycosin-7-*O*-β-D-glucoside, derived from *A. membranaceus* possess a variety of biological activities, such as antioxidant, anti-apoptosis, and anti-inflammatory activities, that reverse ischemic injury (Fu et al., 2014; Li et al., 2017; Yin et al., 2019). In particular, astragaloside IV promotes hippocampal neurogenesis in the intact brain, and astragaloside VI induces proliferation of neural stem cells (NSCs) in the subventricular zone (SVZ) and peri-infarct cortex via activating the epidermal growth factor receptor (EGFR)/mitogen-activated protein kinase (MAPK) pathway after a stroke (Huang et al., 2018; Chen et al., 2019). Hydroxysafflor yellow A is a bioactive ingredient extracted from *C. tinctorius*. It has been reported that hydroxysafflor yellow A improves synaptic plasticity and cognitive function in cerebral ischemia-reperfused rats (Yu et al., 2018). A recent *in vitro* study reported that hydroxysafflor yellow A inhibited apoptosis and autophagy of NSCs via the p38 MAPK/MK2/Hsp27-78 signaling pathway (Li et al., 2019). Paeoniflorin, the main ingredient of *P. lactiflora*, possesses anti-inflammatory activity and improves regional cerebral blood flow and increases neurogenesis following cerebral ischemia (Zhang L.G. et al., 2017; Ko et al., 2018). Taken together, these findings suggested that XSEC exhibits neuroprotective and neurorestorative effects on stroke through multiple components aimed at multiple targets and mechanisms. Further studies on its active components to augment axonal regeneration after a stroke may be of great importance to elucidate the mechanistic basis on XSEC.

Secondly, the exact mechanism by which XSEC and EE restore brain structure and function is still unclear. Our previous study showed that XSEC + EE combination improved neurobehavioral performance by promoting focal neurogenesis and angiogenesis (Zhan et al., 2019). In this study, we further demonstrated that XSEC + EE combination conferred brain remodeling by promoting endogenous oligodendrogenesis and axonal reorganization and downregulating axonal growth inhibitory signaling, thereby improving neurological functional outcome after an ischemic stroke. These findings raised the interesting possibility that the beneficial effect of the combination treatment with XSEC and EE might be related to augmenting endogenous restorative mechanisms and improving global neural environment in the ischemic regions. Future investigations to elucidate the underlying molecular mechanisms that lead to facilitated rehabilitation with XSEC + EE combination treatment are necessary.

## Conclusion

Taken together, our data implicated that XSEC + EE combination had synergistic effects in promoting endogenous brain remodeling by inducing oligodendrogenesis, increasing myelinated axons, and promoting axonal reorganization in the peri-infarct regions coupled with downregulating intrinsic axonal growth inhibitory signaling, thereby improving learning and memory function outcome after an ischemic stroke. This is an important result that should impact the future design of neurorestorative treatment to stimulate brain remodeling processes using multifactorial combination therapies.

## Data Availability Statement

The raw data supporting the conclusions of this article will be made available by the authors, without undue reservation, to any qualified researcher.

## Ethics Statement

The animal study was reviewed and approved by the National Institutes of Health Guide for the Care and Use of Laboratory Animals Capital Medical University Animal Ethics Committee.

## Author Contributions

M-ZL performed qRT-PCR experiments, analyzed data, and drafted the manuscript. X-FF carried out animal experiments. TZ performed behavior tests. YZ contributed to immunostaining examination. LY conducted western blot detection. J-FL performed MRI experiments. H-YZ helped to analyze data. LW revised the manuscript. HZ designed the study, supervised the whole project and reviewed the manuscript.

## Conflict of Interest

The authors declare that the research was conducted in the absence of any commercial or financial relationships that could be construed as a potential conflict of interest.

## Footnotes

1 https://www.slicer.org/
